# Promoter Engineering Reveals the Importance of Heptameric Direct Repeats for DNA Binding by Streptomyces Antibiotic Regulatory Protein–Large ATP-Binding Regulator of the LuxR Family (SARP-LAL) Regulators in Streptomyces natalensis

**DOI:** 10.1128/AEM.00246-18

**Published:** 2018-05-01

**Authors:** Eva G. Barreales, Cláudia M. Vicente, Antonio de Pedro, Javier Santos-Aberturas, Jesús F. Aparicio

**Affiliations:** a Area of Microbiology, Faculty of Biology, University of León, León, Spain; McMaster University

**Keywords:** antifungal agent, gene regulation, LuxR, PAS domain, polyene macrolide, SARP-LAL regulator, Streptomyces

## Abstract

The biosynthesis of small-size polyene macrolides is ultimately controlled by a couple of transcriptional regulators that act in a hierarchical way. A Streptomyces antibiotic regulatory protein–large ATP-binding regulator of the LuxR family (SARP-LAL) regulator binds the promoter of a PAS-LuxR regulator-encoding gene and activates its transcription, and in turn, the gene product of the latter activates transcription from various promoters of the polyene gene cluster directly. The primary operator of PimR, the archetype of SARP-LAL regulators, contains three heptameric direct repeats separated by four-nucleotide spacers, but the regulator can also bind a secondary operator with only two direct repeats separated by a 3-nucleotide spacer, both located in the promoter region of its unique target gene, *pimM*. A similar arrangement of operators has been identified for PimR counterparts encoded by gene clusters for different antifungal secondary metabolites, including not only polyene macrolides but peptidyl nucleosides, phoslactomycins, or cycloheximide. Here, we used promoter engineering and quantitative transcriptional analyses to determine the contributions of the different heptameric repeats to transcriptional activation and final polyene production. Optimized promoters have thus been developed. Deletion studies and electrophoretic mobility assays were used for the definition of DNA-binding boxes formed by 22-nucleotide sequences comprising two conserved heptameric direct repeats separated by four-nucleotide less conserved spacers. The cooperative binding of PimR^SARP^ appears to be the mechanism involved in the binding of regulator monomers to operators, and at least two protein monomers are required for efficient binding.

**IMPORTANCE** Here, we have shown that a modulation of the production of the antifungal pimaricin in Streptomyces natalensis can be accomplished via promoter engineering of the PAS-LuxR transcriptional activator *pimM*. The expression of this gene is controlled by the Streptomyces antibiotic regulatory protein–large ATP-binding regulator of the LuxR family (SARP-LAL) regulator PimR, which binds a series of heptameric direct repeats in its promoter region. The structure and importance of such repeats in protein binding, transcriptional activation, and polyene production have been investigated. These findings should provide important clues to understand the regulatory machinery that modulates antibiotic biosynthesis in Streptomyces and open new possibilities for the manipulation of metabolite production. The presence of PimR orthologues encoded by gene clusters for different secondary metabolites and the conservation of their operators suggest that the improvements observed in the activation of pimaricin biosynthesis by Streptomyces natalensis could be extrapolated to the production of different compounds by other species.

## INTRODUCTION

PAS-LuxR regulators are highly conserved transcriptional factors that combine an N-terminal PAS sensory domain (1) with a C-terminal helix-turn-helix (HTH) motif of the LuxR type for DNA binding (2). The archetype of this class of regulators, PimM, was first identified in the antifungal pimaricin biosynthetic gene cluster from Streptomyces natalensis (3). It was first characterized as a transcriptional activator of pimaricin biosynthesis, as antifungal production was abolished upon gene deletion, and later, its mode of action was characterized at the molecular level (4).

The PimM paradigm is particularly attractive because orthologous proteins are encoded by every polyene cluster and all are able to restore pimaricin production in S. natalensis Δ*pimM* mutants, indicating that these proteins are functionally equivalent to the extent that they are interchangeable (5, 6). This attractiveness multiplies if we take into account that the heterologous expression of these regulators has been successfully used to trigger the production of several polyene macrolides in different producing strains (5). However, the extraordinary thing about PimM is that it can bind a large number of operators external to clusters of polyene biosynthesis, thereby controlling the expression of a large number of nonpolyene genes and hence the processes in which these are involved (7).

The hierarchical superior of *pimM* in the control of pimaricin biosynthesis in S. natalensis is the regulator PimR, a peculiar transcriptional activator that combines an N-terminal *S*treptomyces antibiotic regulatory protein (SARP) DNA-binding domain with a C-terminal half homologous to guanylate cyclases and large ATP-binding regulators of the LuxR family (LAL) (8). The C-terminal half includes the ATP/GTP-binding domain characteristic of these protein families but lacks the characteristic signature sequence at the N terminus of guanylate cyclases or the LuxR-type helix-turn-helix (HTH) motif for DNA binding present at the C terminus of LAL regulators (9). PimR binds a main operator that contains three heptameric direct repeats of the consensus CGGCAAG with 4-bp spacers (10). Such an operator lies in the promoter region of *pimM*, whose expression is activated upon PimR binding, although unlike other SARP operators (11), it does not overlap the −35 promoter box. DNase I protection studies also revealed that PimR binds a secondary operator, with two heptameric repeats of the consensus separated by a 3-bp spacer (10), thus forming 10-bp repeating units instead of the classical 11-bp SARP binding sequences (12).

Interestingly, the binding sequence of PimR (TGGCAAGAAAGCGGCAGGTGTTCGGCAAG [the heptameric repeats are underlined]) is exactly conserved in the intergenic region between *scnRII* and *scnRI* in the *scn* gene cluster of Streptomyces chattanoogensis (*pimM* and *pimR* counterparts, respectively [13]) and also between *pteF* and *pteR* and between *filF* and *filR*, the corresponding counterparts in the filipin gene clusters of Streptomyces avermitilis and Streptomyces filipinensis (14, 15), including the interheptamer nucleotides. Furthermore, the secondary operator is also conserved in these strains, showing almost identical heptameric direct repeats and spacers. PimR, ScnRI, PteR, and FilR are all SARP-LAL regulators of small polyene macrolide biosynthesis, and it is conceivable that their target sequences originated as the result of horizontal transfer. Noteworthy, the consensus heptamer for PimR is also identical to those of SanG (16) and PolR (17), which are SARP-LAL regulators of the biosynthesis of peptidyl nucleoside antibiotics such as nikkomycins (SanG) and polyoxins (PolR); however, in these cases, only two heptameric repeats are present in the operator.

Since PimR is the archetype of SARP-LAL regulators, it was of great interest to characterize the role of those conserved heptameric repeats in the process of transcriptional activation.

## RESULTS

### Promoter engineering and experimental approach.

**FIG 1:**
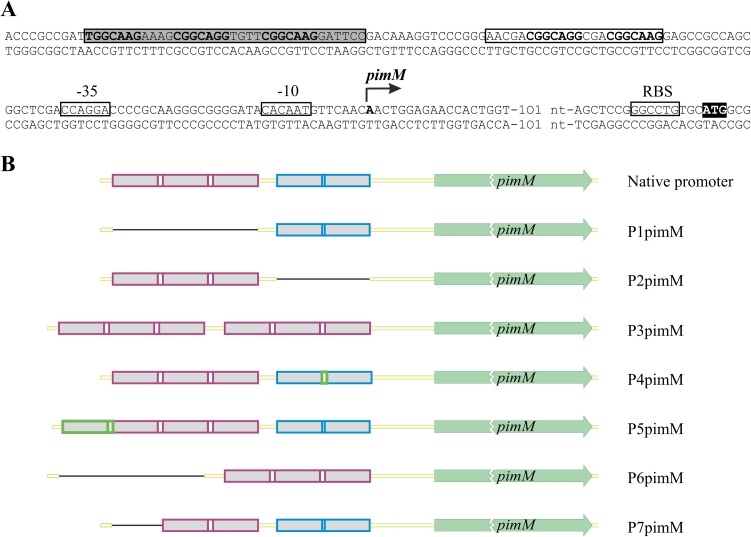
*pimM* promoter and schematic representation of designed *pimM* promoter variants. (A) The transcription start site is indicated by a bent arrow, and the putative −10 and −35 hexanucleotides are boxed. The start codon is shown in a black box. The main protected nucleotide sequence is indicated with a shaded box, and the secondary group of protected nucleotides is boxed without shading. The heptameric repeats are indicated in bold. Data have been extracted from reference 10. (B) Heptameric repeats are shown in gray and spacers in white. The main operators are boxed in magenta, while the secondary operators are boxed in blue. Eliminations of heptamers are indicated by thin black lines and insertions in green. Sizes are not proportional.

To determine the importance of the conserved heptanucleotide repeats on *pimM* expression, we designed seven constructs containing the *pimM* gene under the control of different versions of its promoter (see Materials and Methods) (Fig. 1). The primers used for promoter engineering are indicated in Table 1. In the first of these, called P1pimM, we removed completely the heptanucleotide triplet that constituted the main PimR operator and replaced it with neutral nucleotides (see Materials and Methods). Similarly, in P2pimM, the two direct repeats of the heptamer which formed the secondary operator were eliminated by substitution with neutral nucleotides. In the P3pimM construct, we replaced the secondary operator with a second triplet copy, while in its derivative, P6pimM, the original triplet was removed (Fig. 1B). The P4pimM construct included an extra nucleotide in the doublet spacer, matching it to the triplet spacers. In P5pimM, a further heptamer was inserted before the triplet, whereas in P7pimM, the first heptamer of the main operator was deleted (Fig. 1B). The promoter constructs were designed in a way that the distances between the putative operators and the *pimM* start triplet were not altered (Fig. 1).

**TABLE 1:** Primers used for promoter engineering

Name	Sequence (5′→3′)	Construction
PPM1F	CGACCCGCCGATTGGTACATACATACATACATACATACAAGGATTCCGACAAAGG	P1pimM
PPM1R	CCTTTGTCGGAATCCTTGTATGTATGTATGTATGTATGTACCAATCGGCGGGTCG	P1pimM, P6pimM
PPM2F	CGGGAACGACGGTACATACATACAAGGAGCCGCCAGCG	P2pimM
PPM2R	CGCTGGCGGCTCCTTGTATGTATGTACCGTCGTTCCCG	P2pimM
PPM3F	GGTCCCGGGAACGTGGCAAGAAAGCGGCAGGTGTTCGGCAAGGATTCCACCAGGACCC	P3pimM
PPM34R	CGTTCCCGGGACCTTTGTC	P3pimM, P4pimM
PPM4F	GTCCCGGGAACGACGGCAGGCGAACGGC	P4pimM
PPM5F	GCGCCTTCCCGGCTGGCTGGCAAGAAAGTGGCAAGAAAGCGGCAGGTGTTCG	P5pimM
PPM5R	GCCAGCCGGGAAGGCGCTTTC	P5pimM
PPM6F	CAAAGGTCCCGGGAACGATGGCAAGAAAGCGGCAGGTGTTCGGCAAGGATTCCCCAGGACCC	P6pimM
PPM7F	GCGACCCGCCGATTACATACAAAGCGGCAGGTGTTCGGCAAGG	P7pimM
PimM-F	CGGTCCCCGTGCCTCTCGC	All except P7pimM
PimM-R	CGGCGAGGAGGCTTCAGC	All

The constructs were then cloned into the vector pSET152 (18) for further introduction by conjugation into S. natalensis Δ*pimM*, a strain deficient in the production of pimaricin (3).

### Effects of modification of PimR transcriptional target on pimaricin production.

Exconjugants containing *pimM* under the control of the different versions of the original promoter were fermented for 96 h in yeast extract-malt extract (YEME) medium without sucrose. Samples were obtained every 24 h for growth and pimaricin production measurements. S. natalensis Δ*pimM* containing pSETpimM, a pSET152 derivative with *pimM* under the control of its native promoter, was used as a positive control (3). The same strain with the empty plasmid was used as a negative control.

**FIG 2:**
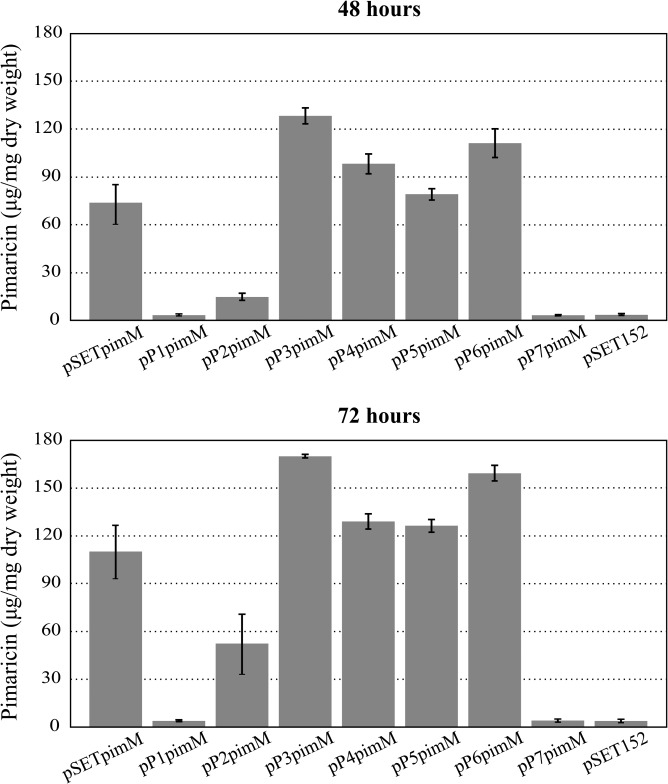
Quantification of pimaricin production in S. natalensis Δ*pimM* after the introduction of the *pimM* gene under the control of different versions of its promoter. Fermentations were carried out in YEME medium without sucrose. S. natalensis Δ*pimM*::pSETpimM was used as the positive control, and S. natalensis Δ*pimM*::pSET152 was the negative control. Data (expressed as μg of pimaricin per mg of dry weight) are the averages from three flasks. Vertical bars indicate the standard deviation values. All the strains showed similar growth profiles. Plasmid names are indicated below the pictures.

All strains showed similar levels of growth, reaching the maximum at 72 h of cultivation (∼2.85 mg/ml), after which a decrease in biomass was observed up to 96 h, probably due to nutrient depletion and the onset of cell death processes. However, significant differences were observed in pimaricin production (Fig. 2). The strains containing the pP1pimM and pP7pimM constructs showed no pimaricin production, whereas pP2pimM restored it to 47% of the positive-control strain. Interestingly, all the remaining promoters tested resulted in an increase in pimaricin production compared to that from the native promoter. While pP4pimM and pP5pimM slightly increased the yields (117% and 114%, respectively, at 72 h of culture), the strains containing the pP3pimM and pP6pimM constructs rendered yields of pimaricin substantially higher than those rendered by the control (154% and 145%, respectively) (Fig. 2).

### Contribution of heptameric repeats to *pimM* transcription.

To study the effect of promoter mutations on *pimM* transcription, a quantitative analysis of *pimM* expression in the different recombinant strains was performed. Total RNA obtained from 48-h cultures, a condition under which pimaricin is actively produced, was used as the template (19). The transcriptional levels of *pimM* in the different strains were compared with those obtained for the positive-control strain, which was assigned a relative expression value of 1.

**FIG 3:**
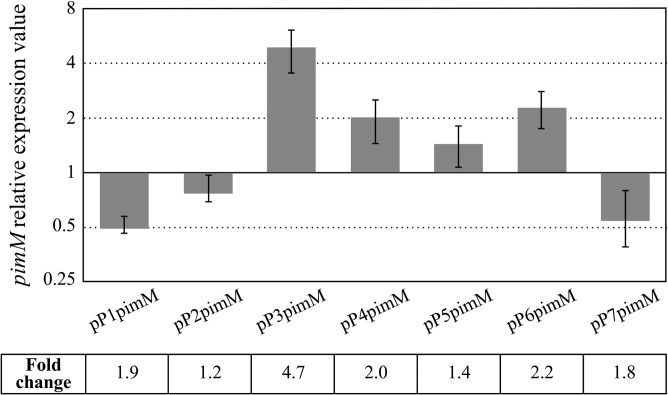
Gene expression analysis of *pimM* under the control of different versions of its promoter. Gene expression was assessed by quantitative RT-PCR with the primers indicated in Materials and Methods. The expression values are relative to 1, the assigned relative value for the expression of *pimM* in S. natalensis Δ*pimM*::pSETpimM. The expression of *rrnA1* (encoding 16S rRNA) was used as the control. Error bars were calculated by measuring the standard deviations of the ratio values from two biological and three technical replicates of each sample. The RNA templates were from 48-h cultures grown in YEME medium without sucrose. Fold change values are indicated.

The differences in *pimM* expression among the different strains were in agreement with the differences in pimaricin production observed in the fermentations (Fig. 2 and 3). Thus, *pimM* expression was severely reduced when we replaced its native promoter with the P1pimM or P7pimM promoter (1.9 or 1.8 times less, respectively), in concordance with the absence of pimaricin production (Fig. 2). For its part, P2pimM reduced the expression of the gene by 20%, accordingly with the reduction observed in pimaricin production (Fig. 2 and 3). The remaining promoter versions rendered an increase in *pimM* expression compared to that in the control, ranging from 140% for P5pimM to 470% for P3pimM. The expression of *pimM* from the P4pimM and P6pimM promoters was approximately double that of the native promoter (Fig. 3). In all cases, the increase in *pimM* expression corresponded to an increase in antifungal production, reaching maxima of expression and production with P3pimM under the conditions tested.

To ascertain that the low expression value observed with P1pimM or P7pimM was not the result of a complete absence of transcripts, endpoint PCR was performed. The results confirmed that transcription was at a low level (not shown).

Except for those with P3pimM, the transcription values showed good concordance with pimaricin production. Figure S2 in the supplemental material shows the correlation between both data, including a least-square line fit that resulted in a Pearson's correlation coefficient (*r*^2^) of 0.8918 for the plot.

### Effects on DNA binding.

To corroborate the above results, we checked the ability of the PimR SARP domain to bind the different promoter versions. For that purpose, we performed electrophoretic mobility shift assays (EMSAs) with GST-PimR^SARP^ (10) and DNA probes containing the different promoters.

**FIG 4:**
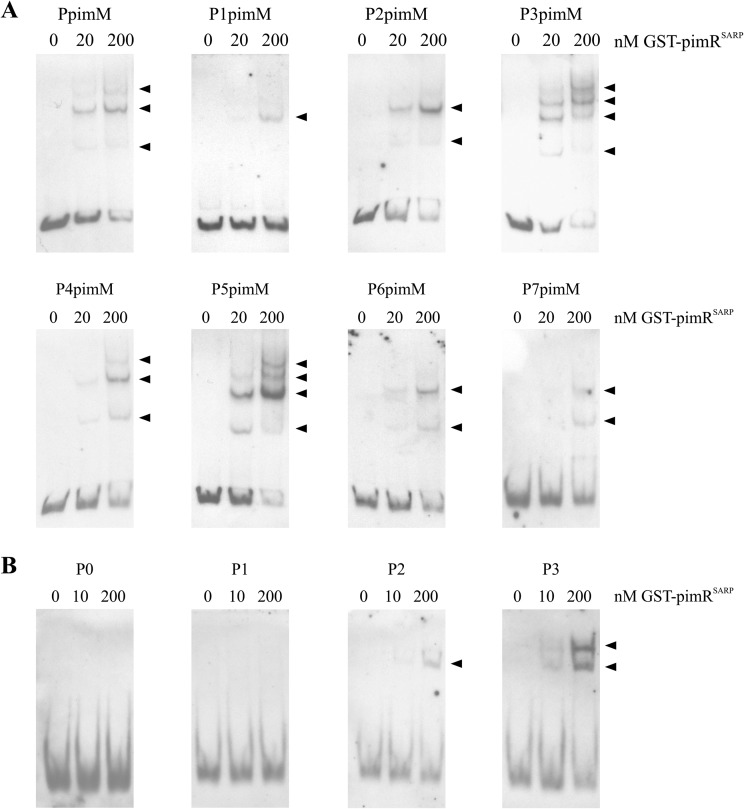
EMSAs of GST-PimR^SARP^ binding to different putative promoter regions (A) or synthetic DNA duplexes (B). In panel A, the promoter names are indicated above the pictures. In panel B, DNA duplexes contain zero (P0), one (P1), two (P2), or three (P3) heptamers. Base compositions of the duplexes are indicated in Table 2. The arrowheads indicate the DNA-protein complexes. All experiments were carried out with 2 ng labeled DNA probe. Left lane, control without protein; right lanes, increasing concentrations (10 to 200 nM) of GST-PimR^SARP^ protein.

Figure 4 shows the results from EMSAs performed with increasing concentrations of GST-PimR^SARP^. To discard the possibility that interactions could be produced by the GST moiety of the fusion protein, control reactions were performed under the same conditions but using pure GST (10 μM) instead of GST-PimR^SARP^. This experiment produced negative results in all cases, excluding the possible binding of the GST protein to the promoters (not shown). In the cases where retarded bands were observed, the intensity of the band(s) was diminished by the addition of the same unlabeled DNA (not shown), suggesting that binding is specific.

Interestingly, the number of shifted bands observed with the different variants of the promoter was in agreement with the binding of a protein monomer to each of the heptamers, with a minimum requirement of two protein monomers for efficient binding (see below). Thus, the native promoter would be able to accommodate two or three monomers in the region containing the triplet of heptamers plus two in the doublet, and three retardation bands were observed (Fig. 4A). Similarly, P4pimM, which would enable the same number of binding complexes, yielded three shifted bands. With the P1pimM promoter, only one retardation band was observed, in accordance with the presence in this promoter of only two heptamers that enable the binding of two protein monomers. P2pimM and P6pimM each contain one triplet of heptamers, thus enabling the formation of two binding complexes and yielding two shifted bands. P3pimM, with two triplets of heptamers, and P5pimM, with a quadruplet and a doublet of heptamers, both enabled the formation of 4 binding complexes, and four shifted bands were produced in both cases (Fig. 4A).

In general, there was a clear correlation between GST-PimR^SARP^ binding to the different probes and the pimaricin production observed with the different promoters. Thus, while little interaction was observed with the probes containing P1pimM or P7pimM, strong band shifting was observed with the probes containing promoters that yielded the highest pimaricin production, such as P3pimM and P6pimM.

Altogether, these results indicate that the differences observed in *pimM* transcription could be the consequence of *pimR* binding to the different promoter versions and the subsequent recruitment of RNA polymerase.

### Two heptameric repeats are required for PimR binding.

To confirm our previous interpretation of DNA-binding results, we constructed different duplex DNA probes using the triplet of heptamers in the native promoter as a model (Table 2). Thus, P3 contained the triplet of heptamers, P2 contained only two heptamers, P1 contained just one heptamer, and P0 contained no heptamer. The binding of GST-PimR^SARP^ to the duplexes was then studied by EMSAs. Interestingly, the results showed that two heptameric repeats are necessary for GST-PimR^SARP^ binding (Fig. 4B). While there is a clear binding of GST-PimR^SARP^ to probe P3 containing the triplet of heptamers (forming two complexes with different mobilities, as expected), only one retardation band was observed with probe P2 (containing two heptamers), and no binding was observed with probe P1 (one heptamer) or P0 (without any heptamer) under the same reaction conditions.

**TABLE 2:** Oligonucleotides used for EMSAs

Primer	Sequence (5′→3′)	Probe
P0-PimR-F	GGCTGGCGACCCGCCGATTACATACATACATACATACATACATACAGGATTCCGACAAAGGTCCCGGGA	P0
P0-PimR-R	TCCCGGGACCTTTGTCGGAATCCTGTATGTATGTATGTATGTATGTATGTAATCGGCGGGTCGCCAGCC	P0
P1-PimR-F	GGCTGGCGACCCGCCGATTACATACATACCGGCAAGTGTTCATACAGGATTCCGACAAAGGTCCCGGGA	P1
P1-PimR-R	TCCCGGGACCTTTGTCGGAATCCTGTATGAACACTTGCCGGTATGTATGTAATCGGCGGGTCGCCAGCC	P1
P2-PimR-F	GGCTGGCGACCCGCCGATTACATACAAAGCGGCAGGTGTTCGGCAAGGATTCCGACAAAGGTCCCGGGA	P2
P2-PimR-R	TCCCGGGACCTTTGTCGGAATCCTTGCCGAACACCTGCCGCTTTGTATGTAATCGGCGGGTCGCCAGCC	P2
P3-PimR-F	GGCTGGCGACCCGCCGATTGGCAAGAAAGCGGCAGGTGTTCGGCAAGGATTCCGACAAAGGTCCCGGGA	P3
P3-PimR-R	TCCCGGGACCTTTGTCGGAATCCTTGCCGAACACCTGCCGCTTTCTTGCCAATCGGCGGGTCGCCAGCC	P3

## DISCUSSION

PAS-LuxR regulator-encoding genes are associated with several polyene macrolide biosynthetic gene clusters, thus constituting a possible landmark of this type of gene cluster. In this context, they are transcriptional activators essential for the biosynthesis of the polyene encoded within the cluster. Their expression is a bottleneck in the biosynthesis of the antifungal; thus, polyene production is easily increased upon gene dosage increase (20, 21). Here, we have shown that another strategy for production increase is to optimize its promoter for more efficient RNA polymerase recruitment. The SARP-LAL regulator PimR is hierarchically superior to the PAS-LuxR regulator PimM and activates the transcription of the latter. PimR binds a main operator that contains three heptameric direct repeats and a second operator with two copies of the repeat (10). As shown here, the two operators contribute differently to *pimM* expression.

The three direct heptameric repeats have proven to be important for the transcription of *pimM* and essential for the subsequent pimaricin biosynthesis. Thus, in the S. natalensis Δ*pimM* cultures containing pP1pimM or pP7pimM constructs, where the entire triplet of heptamers or just one of the heptamers is removed, the production of the polyene was not detected, whereas the *pimM* transcript level was approximately half that of the control strain. Such a reduction in *pimM* transcription turned out to be sufficient to completely abolish pimaricin production by S. natalensis. In S. natalensis Δ*pimM*::pP2pimM, which lacked the doublet of heptamers, the expression of the PAS-LuxR regulator decreased by 20%, whereas pimaricin production was reduced by ca. 50% compared to that of the strain containing the native promoter, indicating that the doublet is less relevant than the triplet. However, the addition of one heptamer to the triplet (P5pimM) did not substantially improve pimaricin production compared with that from the native promoter. This suggests that the optimum number of repeats of the heptamer recognized by PimR is three and that at least one triplet is required at the operator to activate *pimM* transcription.

In addition, the size of the spacers between heptamers is also an influential factor in *pimM* transcription, with four nucleotides preferable to three, as demonstrated by the increases in *pimM* expression and pimaricin production by the strain S. natalensis Δ*pimM*::pP4pimM compared to those of the control with the native promoter. The classical SARP binding sequence is 11 bp in length (12) (one complete turn of the DNA helix), which is equivalent to one heptamer and a four-nucleotide spacer. Because the repeats are separated by 11 nucleotides, SARP regulator monomers are thought to bind the same face of the DNA (11), presumably in a cooperative way that helps in the recognition of adjacent repeats (11, 22), and this could also be the case for SARP-LAL regulatory proteins. But unlike SARP regulators which bind DNA overlapping the −35 hexamer of target promoters, just on the opposite face of the helix to which RNA polymerase binds, the SARP-LAL regulator binding sequence is located upstream of that hexamer. Furthermore, DNase I protection studies have shown that PimR binds the heptameric repeats on the *pimM* coding strand (10) and therefore on the same face of the helix to which RNA polymerase binds. This is in agreement with a class I activation mechanism, where PimR would contact the C-terminal domain of the RNA polymerase α subunit, resulting in the recruitment of the RNA polymerase to the *pimM* promoter (23).

The location of the triplet also appears to play an important role in the activation of transcription by PimR. S. natalensis Δ*pimM*::pP6pimM, which contained just the triplet located at the position of the doublet, prompted *pimM* transcription and polyene production to substantially greater extents than those prompted by the native promoter. Accordingly, the presence of two triplets in the promoter (replacement of the doublet by a second triplet; pP3pimM) was the modification that rendered the best results, increasing *pimM* transcription almost 5-fold and polyene production by 50%.

Our results from EMSAs are in agreement with the binding of protein monomers in a cooperative way that helps in the recognition of adjacent repeats. Thus, a protein monomer would bind to each of the heptamers (and their four-nucleotide spacers), with a minimum requirement of two protein monomers for efficient binding. One heptamer is not sufficient for sustained PimR binding, and at least two heptameric repeats are required for proper binding. A similar mode of binding has been proposed for other proteins belonging to the SARP family, including AfsR, a global regulator of secondary metabolism in Streptomyces coelicolor that consists of an N-terminal SARP domain, a central ATPase domain, and a C-terminal tetratricopeptide repeat (11), or DnrI, a small-size SARP involved in daunorubicin biosynthesis (22). This cooperative model of binding has also been suggested for the Escherichia coli transcription factor OmpR (24). SARPs and OmpR share the same DNA-binding domain architecture (12), and all are thought to bind DNA as monomers, with two monomers required for productive binding (11, 24). Additionally, the reduced transcription observed in promoter versions with no triplet of heptamers, such as P1pimM and P7pimM, and the absence of pimaricin production in both cases suggest that at least three monomers are required to efficiently recruit RNA polymerase.

**FIG 5:**
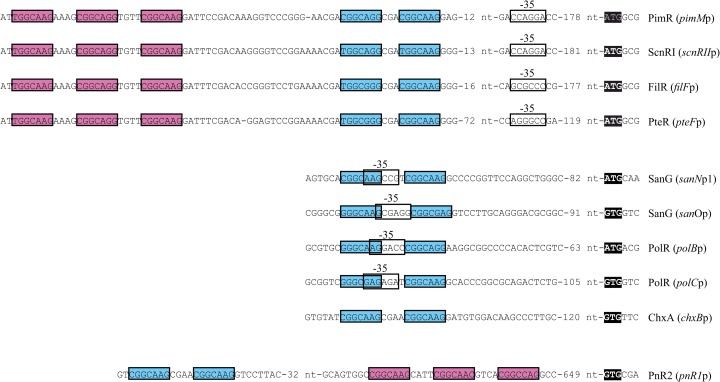
Alignment of PimR operators and those of its counterparts. The heptameric repeats of the main operator sequences are boxed in magenta, and those of the secondary operators are boxed in blue. The −35 hexamers of characterized promoters are boxed in white. The start codons are shown in black boxes. The alignment includes the target operators of PimR and the putative operators of orthologous polyene macrolide regulators such as PteR from S. avermitilis, FilR from S. filipinensis, or ScnRI from S. chattanoogensis. The putative operators of the phoslactomycin activator PnR2 from S. platensis SAM-0654, the cycloheximide regulator ChxA from Streptomyces sp. YIM56141, and the operators of peptidyl nucleoside activators PolR and SanG are also included.

We previously proposed that the same domain arrangement between PimR and its counterparts SanG and PolR could be related to the detection and response to signals triggering the production of antifungals and that it was plausible that these regulators with highly similar architectures could share similar regulatory mechanisms (10). Our results now indicate that there are some differences between the mechanisms of recognition of operators by SARP-LAL regulators of peptidyl nucleosides (SanG and PolR) and those of polyenes. While the target operators of SanG and PolR have heptamer doublets (16, 17), PimR requires at least one heptanucleotide triplet to efficiently activate *pimM* transcription. Due to the high similarity among ScnRI, PteR, FilR and PimR, as well as the high degree of conservation of their corresponding operators (Fig. 5), it is expected that these orthologues also need triplets of the consensus heptanucleotide for transcription activation. Besides that, peptidyl nucleoside regulators operators overlap the −35 hexamers of target promoters, while the operators of polyene SARP-LALs do not (Fig. 5).

Recently, another two PimR orthologues have been described in gene clusters for the biosynthesis of antifungal compounds: PnR2, a transcriptional activator of phoslactomycin biosynthesis in Streptomyces platensis strain SAM-0654 (25), and ChxA, from the cycloheximide cluster in Streptomyces sp. strain YIM56141 (26). In both cases, the SARP-LAL regulatory gene is transcribed divergently from the gene located upstream, although the function of this gene is not conserved. *In silico* analyses of these promoter regions enabled the detection of putative PimR operators: a triplet and a doublet of the consensus heptanucleotide in the case of PnR2 and a doublet in the case of ChxA, all with 4-bp spacers (Fig. 5). These observations suggest that the mode of action of PnR2 would be more similar to that described for PimR than the one expected for SanG or PolR, whereas that of ChxA would be just the opposite. The presence of the 4-bp spacers in the doublet recognized by PnR2 tempts us to speculate that it would be a more efficient operator than the PimR one, where the introduction of an extra nucleotide to the 3-bp spacer doubled *pimM* transcription.

The findings reported here should provide important clues to understanding the regulatory machinery that modulates antibiotic biosynthesis in Streptomyces. The presence of PimR orthologues encoded by gene clusters for different secondary metabolites and the conservation of their operators suggest that the improvements observed in the activation of pimaricin biosynthesis by S. natalensis could be extrapolated to the production of different compounds by other species.

## MATERIALS AND METHODS

### Strains and cultivation.

S. natalensis Δ*pimM* (3) was routinely grown in YEME medium (27) without sucrose. Sporulation was achieved in tomato paste-baby oatmeal (TBO) medium (28) at 30°C. Escherichia coli strain DH5α was used as a host for DNA manipulation. E. coli BL21(DE3) was used for expression studies. E. coli ET12567::pUZ8002 was used as donor in intergeneric conjugations.

### Plasmids and DNA manipulation procedures.

Intergeneric conjugation between E. coli ET12567::pUZ8002 and S. natalensis Δ*pimM* was performed as described previously (29). pUC19 (New England BioLabs) was used as the routine cloning vector, pSET152 (Am^r^, pUC18 replicon, ϕC31 *attP* [18]) was used for intergeneric conjugations, and pPimR^SARP^ was the vector used for protein expression (10). Plasmid DNA preparation, DNA digestion, fragment isolation, and transformation of E. coli were performed with standard procedures. PCRs were carried out using hybrid DNA polymerase as described by the enzyme supplier (EURx). DNA sequencing was accomplished by the dideoxynucleotide chain-termination method using the DYEnamic ET terminator cycle sequencing kit (GE Healthcare) with an Applied Biosystems ABI 3130XL DNA genetic analyzer (Foster City, CA, USA).

### Construction of promoter versions.

All constructs were obtained by fusion PCR. The primers are indicated in Table 1. For P1pimM to P5pimM, the primer pairs used for the individual PCRs were PimM-F/PPMX-R and PPMX-F/PimM-R, where X was the version number of the promoter to be obtained, and S. natalensis genomic DNA (gDNA) was used as the template. In the case of P6pimM, one of the pairs was PimM-F/PPM1-R and the other one was PPM6-F/PimM-R. Using as the template the purified fragments from these first two PCRs, we performed fusion PCR with the PimM-F/PimM-R oligonucleotides to obtain the *pimM* gene together with its terminator. In all cases, the mutations were introduced into the sequences of the primers so that they were conserved in the fusion PCR product. In contrast to the above cases, P7pimM was obtained by a single PCR with the primers PPM7-F and PimM-R, which gave rise to a 987-bp fragment containing *pimM* under the control of a promoter lacking the first heptamer of the triplet.

All the constructs obtained were cloned into the EcoRV site of pSET152, checked by sequencing, and transformed into the nonmethylating strain E. coli ET12567::pUZ8002. One of the transformants was selected and introduced into S. natalensis Δ*pimM* by conjugation as described previously (29). Exconjugants were selected by their apramycin resistance phenotype and were checked by PCR with primers internal to the plasmid.

The removal of heptameric repeats was performed by replacing the nucleotides of the heptamer with a sequence of neutral nucleotides not recognized by PimR. In this way, the distance between the putative operator and the *pimM* start triplet was not altered. Table S1 in the supplemental material shows the complete sequences of the designed promoters.

### Assessment of pimaricin production.

To assay pimaricin in culture broths, 1 ml of culture was extracted with 4 ml of methanol and further diluted with methanol to bring the absorbance at 319 nm in the range of 0.1 to 0.4 units. Control solutions of pure pimaricin (Sigma) were used as the control. To confirm the identity of pimaricin, a UV-visible absorption spectrum (absorption peaks at 319, 304, 291, and 281 nm) was routinely determined in a Hitachi U-2900 spectrophotometer. The quantitative determination of pimaricin was performed as previously described (30), using a Mediterranea Sea C_18_ column (4.6 mm by 150 mm; particle size, 3 mm [Teknokroma]).

### Isolation of total RNA.

RNA was extracted as described previously (6). Briefly, 2 ml from liquid cultures in YEME medium without sucrose was harvested by centrifugation and immediately frozen by immersion in liquid nitrogen. The cells were resuspended in lysis solution (600 μl RLT buffer [RNeasy minikit; Qiagen], 6 μl 2-mercaptoethanol) and disrupted using a sonicator (Ultrasonic Processor XL; Misonix Inc.). An RNeasy minikit (Qiagen) was used for RNA isolation using an RNase-free DNase set (Qiagen) as specified by the manufacturer, followed by two consecutive digestions with Turbo DNase from Ambion according to the manufacturer's instructions.

### Reverse transcription-PCR.

Transcription was studied by using the SuperScript one-step reverse transcriptase PCR (RT-PCR) system with Platinum *Taq* DNA polymerase (Invitrogen) and 150 ng of total RNA as the template. The conditions were as follows: first-strand cDNA synthesis at 45°C for 40 min followed by heating at 94°C for 2 min and amplification consisting of 30 cycles at 94°C for 40 s, 63°C for 30 s, and 72°C for 30 s. Primers 5′-GCCTGCCCGACACGACCG-3′ and 5′-TCCACGACCTCGGGCGGC-3′ were designed to cover the 3′-end region of *pimM*, and 5′-GGGGTGGATTAGTGGCGAACG-3′ and 5′-CAACCCGAAGGCCGTCATCC-3′ were used for 16S rRNA, generating PCR products of 304 bp and 340 bp, respectively. Negative-control reactions were carried out with each set of primers and Platinum *Taq* DNA polymerase in order to confirm the absence of contaminating DNA in the RNA preparations. The identity of each amplified product was corroborated by direct sequencing of the PCR product.

### Reverse transcription-quantitative PCR.

Reverse transcription of total RNA was performed on selected samples with 5 μg of RNA and 12.5 ng/μl of random hexamer primer (Invitrogen) using SuperScript III reverse transcriptase (Invitrogen). All RNA samples were analyzed with the Agilent 2100 Bioanalyzer (Agilent Technologies, Palo Alto, CA), and only those with RNA integrity number (RIN) values (31) ranging from 7.5 to 8.5 were selected. Each reaction was performed in a 20-μl mixture containing SYBR Premix *Ex Taq* (TaKaRa), 200 to 300 nM each primer, and the template cDNA diluted 1:2 and run on a StepOnePlus real-time PCR system (Applied Biosystems). The reactions were carried out in two biological replicates with three technical replicates each, and appropriate controls were included to verify the absence of gDNA contamination in the RNA samples and primer-dimer formation. Primers 5′-TTGGAGAATTGACGCACCAG-3′ and 5′-ATCCAGCAGGCGAACCAG-3′ for *pimM* and 5′-GACGCAACGCGAAGAACC-3′ and 5′-TGCGGGACTTAACCCAACATC-3′ for *rrnA1* were designed to generate PCR products of 125 and 137 bp, respectively, near the 5′ ends of the mRNA. The PCRs were initiated by incubating the sample at 95°C for 10 min followed by 40 cycles at 95°C for 15 s and 66 to 70°C (depending of the set of primers used) for 34 s. To check the specificity of real-time PCRs, a DNA melting curve analysis was performed by holding the sample at 60°C for 60 s, followed by slow ramping of the temperature to 95°C. Baseline and threshold values were determined by the StepOnePlus software. *C_T_* values were normalized with respect to *rrnA1* mRNA (encoding 16S rRNA). The relative changes in gene expression were quantified using the Pfaffl method (32) and REST software (33). The corresponding real-time PCR efficiency (*E*) of one cycle in the exponential phase was calculated according to the equation (*E* = 10^(−1/slope)^ (34) using 5-fold dilutions of genomic DNA ranging from 0.013 to 40 ng (*n* = 5 or 6 with three replicates for each dilution), with a coefficient of determination (*R*^2^) of >0.99 (see Fig. S1).

### DNA-protein binding assays.

DNA binding was tested by using electrophoretic mobility shift assays (EMSAs). The DNA fragments used for EMSAs were amplified by PCR using the primers PMF (5′-CGCTCCTGTGGATCGTTGCATAG-3′) and PMR (5′-TACAGGATCCGTTCTATCAAGGCTCGCCATGCAC-3′) for probes PpimM to P6pimM, or P7F (5′-CGTATGTTGTGTGGAATTGTGAGCG-3′) and PMR for probe P7pimM, using the corresponding construct as the template. The amplification products were then sequenced to confirm the absence of any mutations and then labeled at both ends with a digoxigenin (DIG) oligonucleotide 3′-end labeling kit (2nd generation [Roche Applied Science]). Binding assays were performed with GST-PimR^SARP^ protein as described previously (10).

To obtain DNA duplexes for the validation of the binding site, one oligonucleotide pair (either P0-PimR-F and P0-PimR-R for P0, P1-PimR-F and P1-PimR-R for P1, P2-PimR-F and P2-PimR-R for P2, or P3-PimR-F and P3-PimR-R for P3) (Table 2) was annealed by heating the pair at 95°C for 2 min and then at 65°C for 3 min and later slowly cooled to 25°C at 1°C/min in Tris-EDTA (TE) buffer, followed by PAGE purification and 3′-end labeling.

## Supplementary Material

Supplemental material
