# Supplementary material for: Promoter Engineering Reveals the Importance of Heptameric Direct Repeats for DNA Binding by Streptomyces Antibiotic Regulatory Protein–Large ATP-Binding Regulator of the LuxR Family (SARP-LAL) Regulators in Streptomyces natalensis
Source: Appl Environ Microbiol. 2018 May 1;84(10):e00246-18. doi: 10.1128/AEM.00246-18 (PMC5930380; doi:10.1128/AEM.00246-18)
Supplement: Supplemental material [file supp_84_10_e00246-18__index.html]

Supplemental material 

# Promoter Engineering Reveals the Importance of Heptameric Direct Repeats for DNA Binding by Streptomyces Antibiotic Regulatory Protein–Large ATP-Binding Regulator of the LuxR Family (SARP-LAL) Regulators in Streptomyces natalensis

## Supplemental material

- Supplemental file 1 -

  Primer efficiency calculation (Fig. S1); correlation between relative expression values for the *pimM* gene under the control of different versions of the promoter and pimaricin production (Fig. S2); designed promoter sequences (Table S1).

  PDF, 150K
