# Supplementary material for: Promoter Engineering Reveals the Importance of Heptameric Direct Repeats for DNA Binding by Streptomyces Antibiotic Regulatory Protein–Large ATP-Binding Regulator of the LuxR Family (SARP-LAL) Regulators in Streptomyces natalensis
Source: Appl Environ Microbiol. 2018 May 1;84(10):e00246-18. doi: 10.1128/AEM.00246-18 (PMC5930380; doi:10.1128/AEM.00246-18)
Supplement: Supplemental material [file AEM.00246-18_zam010188499s1.pdf]

Supplementary information for:

Promoter engineering reveals the importance of heptameric direct repeats for DNA-binding by SARP-LAL regulators in Streptomyces

Eva G. Barreales,[a] Cláudia M. Vicente,[a*] Antonio de Pedro,[a] Javier Santos-Aberturas,[a**] Jesús F. Aparicio[a#]

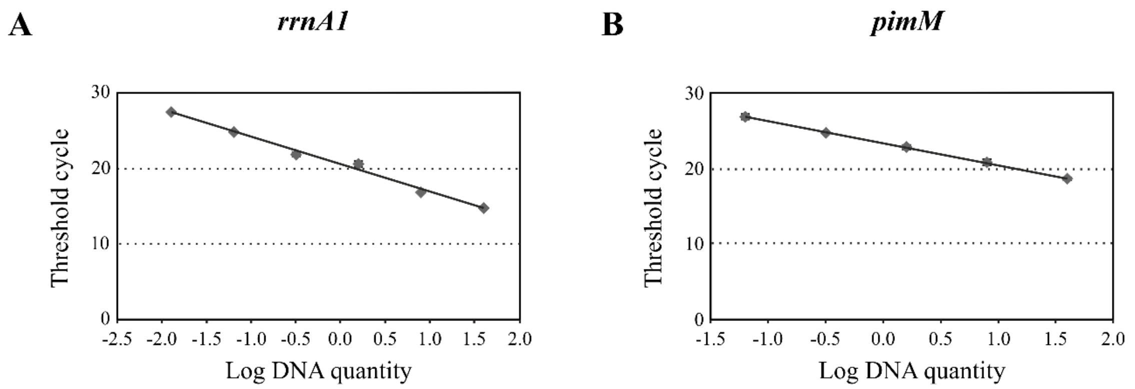

C

Gene	Slope	R^2	Efficiency
rrnA1	-3.6271	0.990	0.887
pimM	-2.9435	0.999	1.186

Fig. S1: Primer efficiency. The efficiency of each set of primers was calculated according to the equation $E = 10^{[-1/slope]}-1$. Using 5-fold dilutions of genomic DNA, the resulting Ct values were plotted against the logarithm of the DNA quantity as shown in A (primers for *rrnA1*), and B (primers for *pimM*). Data are from three replicates, values represent the mean and the vertical bars ± SD. Panel C summarizes information obtained from all plotted data.

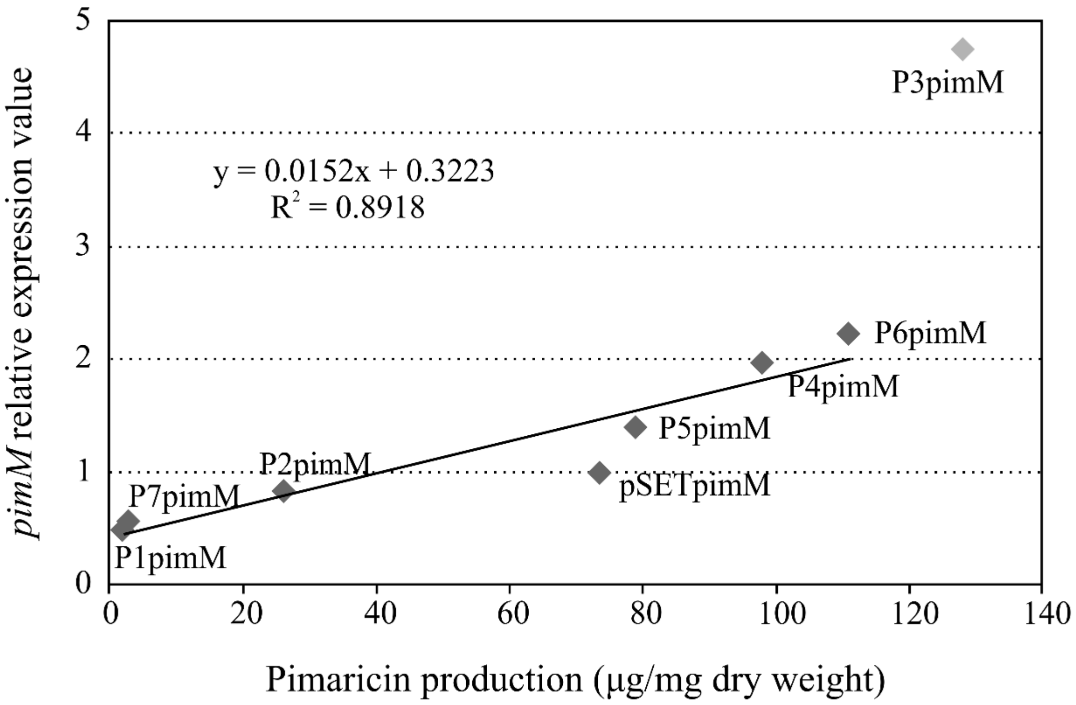

Fig. S2: Correlation between the relative expression value of *pimM* gene under the control of different versions of the promoter and pimaricin production. Gene expression was assessed using RT-qPCR with the primers indicated in material and methods. The relative values are referred to 1, the assigned relative value for the expression of *pimM* in *S. natalensis ΔpimM*::pSETpimM. Specific polyene production values are shown. Similar results were obtained when we compared volumetric production values. A least square straight line fit is also shown.

Table S1: Designed promoter sequences.

Promoter	Sequence (5'→3')
**Native**	CGGTCCCCGTGCCTCTCGCGGATATGGCTGACTGACGGACGGATTCGA<br>GCTGGCTGGCCCCCCGCTCCTGTGGATCGTTGCATAGAGAAAGCGCCT<br>TCCCGGCTGGCGACCCGCCGATTGGCAAGAAAGCGGCAGGTGTTCGG<br>CAAGGATTCCGACAAAGGTCCCGGGAACGACGGCAGGCGACGGCAAG<br>GAGCCGCCAGCGGCTCGACCAGGACCCCGCAAGGGCGGGGATACACA<br>ATGTTCAACAACTGGAGAACCACTGGTCCCAGGGCCGATGACGCCCCT<br>GGTGACGGCGTTCAACGCCGCTTGCCAGCCTCCGAATTGACTTCGCCC<br>TCAAGTTTCGACAGGGAGCCCTCTCTTGCTGAGTCCAGCACCTTCAGC<br>TCCGGGCCTGTGC
**P1**	CGGTCCCCGTGCCTCTCGCGGATATGGCTGACTGACGGACGGATTCGA<br>GCTGGCTGGCCCCCCGCTCCTGTGGATCGTTGCATAGAGAAAGCGCCT<br>TCCCGGCTGGCGACCCGCCGATTGGTACATACATACATACATACATAC<br>AAGGATTCCGACAAAGGTCCCGGGAACGACGGCAGGCGACGGCAAGG<br>AGCCGCCAGCGGCTCGACCAGGACCCCGCAAGGGCGGGGATACACAA<br>TGTTCAACAACTGGAGAACCACTGGTCCCAGGGCCGATGACGCCCCTG<br>GTGACGGCGTTCAACGCCGCTTGCCAGCCTCCGAATTGACTTCGCCCT<br>CAAGTTTCGACAGGGAGCCCTCTCTTGCTGAGTCCAGCACCTTCAGCT<br>CCGGGCCTGTGC
**P2**	CGGTCCCCGTGCCTCTCGCGGATATGGCTGACTGACGGACGGATTCGA<br>GCTGGCTGGCCCCCCGCTCCTGTGGATCGTTGCATAGAGAAAGCGCCT<br>TCCCGGCTGGCGACCCGCCGATTGGCAAGAAAGCGGCAGGTGTTCGG<br>CAAGGATTCCGACAAAGGTCCCGGGAACGACGGTACATACATACAAG<br>GAGCCGCCAGCGGCTCGACCAGGACCCCGCAAGGGCGGGGATACACA<br>ATGTTCAACAACTGGAGAACCACTGGTCCCAGGGCCGATGACGCCCCT<br>GGTGACGGCGTTCAACGCCGCTTGCCAGCCTCCGAATTGACTTCGCCC

	TCAAGTTTCGACAGGGAGCCCTCTCTTGCTGAGTCCAGCACCTTCAGC TCCGGGCCTGTGC
**P3**	CGGTCCCCGTGCCTCTCGCGGATATGGCTGACTGACGGACGGATTCGA GCTGGCTGGCCCCCCGCTCCTGTGGATCGTTGCATAGAGAAAGCGCCT TCCCGGCTGGCGACCCGCCGATTGGCAAGAAAGCGGCAGGTGTTCGG CAAGGATTCCGACAAAGGTCCCGGGAACGTGGCAAGAAAGCGGCAGG TGTTCGGCAAGGATTCCACCAGGACCCCGCAAGGGCGGGGATACACA ATGTTCAACAACTGGAGAACCACTGGTCCCAGGGCCGATGACGCCCCT GGTGACGGCGTTCAACGCCGCTTGCCAGCCTCCGAATTGACTTCGCCC TCAAGTTTCGACAGGGAGCCCTCTCTTGCTGAGTCCAGCACCTTCAGC TCCGGGCCTGTGC
**P4**	CGGTCCCCGTGCCTCTCGCGGATATGGCTGACTGACGGACGGATTCGA GCTGGCTGGCCCCCCGCTCCTGTGGATCGTTGCATAGAGAAAGCGCCT TCCCGGCTGGCGACCCGCCGATTGGCAAGAAAGCGGCAGGTGTTCGG CAAGGATTCCGACAAAGGTCCCGGGAACGACGGCAGGCGAACGGCAA GGAGCCGCCAGCGGCTCGACCAGGACCCCGCAAGGGCGGGGATACAC AATGTTCAACAACTGGAGAACCACTGGTCCCAGGGCCGATGACGCCCC TGGTGACGGCGTTCAACGCCGCTTGCCAGCCTCCGAATTGACTTCGCC CTCAAGTTTCGACAGGGAGCCCTCTCTTGCTGAGTCCAGCACCTTCAG CTCCGGGCCTGTGC
**P5**	CGGTCCCCGTGCCTCTCGCGGATATGGCTGACTGACGGACGGATTCGA GCTGGCTGGCCCCCCGCTCCTGTGGATCGTTGCATAGAGAAAGCGCCT TCCCGGCTGGCTGGCAAGAAAGTGGCAAGAAAGCGGCAGGTGTTCGG CAAGGATTCCGACAAAGGTCCCGGGAACGACGGCAGGCGACGGCAAG GAGCCGCCAGCGGCTCGACCAGGACCCCGCAAGGGCGGGGATACACA ATGTTCAACAACTGGAGAACCACTGGTCCCAGGGCCGATGACGCCCCT GGTGACGGCGTTCAACGCCGCTTGCCAGCCTCCGAATTGACTTCGCCC

	TCAAGTTTCGACAGGGAGCCCTCTCTTGCTGAGTCCAGCACCTTCAGC<br>TCCGGGCCTGTGC
**P6**	CGGTCCCCGTGCCTCTCGCGGATATGGCTGACTGACGGACGGATTCGA<br>GCTGGCTGGCCCCCCGCTCCTGTGGATCGTTGCATAGAGAAAGCGCCT<br>TCCCGGCTGGCGACCCGCCGATTGGTACATACATACATACATACATAC<br>AAGGATTCCGACAAAGGTCCCGGGAACGATGGCAAGAAAGCGGCAGG<br>TGTTCGGCAAGGATTCCCCAGGACCCCGCAAGGGCGGGGATACACAA<br>TGTTCAACAACTGGAGAACCACTGGTCCCAGGGCCGATGACGCCCCTG<br>GTGACGGCGTTCAACGCCGCTTGCCAGCCTCCGAATTGACTTCGCCCT<br>CAAGTTTCGACAGGGAGCCCTCTCTTGCTGAGTCCAGCACCTTCAGCT<br>CCGGGCCTGTGC
**P7**	CCGACCCGCCGATTACATACAAAGCGGCAGGTGTTCGGCAAGGATTCC<br>GACAAAGGTCCCGGGAACGACGGCAGGCGACGGCAAGGAGCCGCCA<br>GCGGCTCGACCAGGACCCCGCAAGGGCGGGGATACACAATGTTCAAC<br>AACTGGAGAACCACTGGTCCCAGGGCCGATGACGCCCCTGGTGACGG<br>CGTTCAACGCCGCTTGCCAGCCTCCGAATTGACTTCGCCCTCAAGTTTC<br>GACAGGGAGCCCTCTCTTGCTGAGTCCAGCACCTTCAGCTCCGGGCCT<br>GTGC
